# Quantum arbitrary waveform generator

**DOI:** 10.1126/sciadv.add4019

**Published:** 2022-10-28

**Authors:** Kan Takase, Akito Kawasaki, Byung Kyu Jeong, Takahiro Kashiwazaki, Takushi Kazama, Koji Enbutsu, Kei Watanabe, Takeshi Umeki, Shigehito Miki, Hirotaka Terai, Masahiro Yabuno, Fumihiro China, Warit Asavanant, Mamoru Endo, Jun-ichi Yoshikawa, Akira Furusawa

**Affiliations:** ^1^ Department of Applied Physics, School of Engineering, The University of Tokyo, 7-3-1 Hongo, Bunkyo-ku, Tokyo 113-8656, Japan.; ^2^ Optical Quantum Computing Research Team, RIKEN Center for Quantum Computing, 2-1 Hirosawa, Wako, Saitama 351-0198, Japan.; ^3^ NTT Device Technology Labs, NTT Corporation, 3-1 Morinosato Wakamiya, Atsugi, Kanagawa 243-0198, Japan.; ^4^ Advanced ICT Research Institute, National Institute of Information and Communications Technology, 588-2 Iwaoka, Nishi-ku, Kobe, Hyogo 651-2492, Japan.; ^5^ Graduate School of Engineering, Kobe University, 1-1 Rokkodai-cho, Nada-ku, Kobe, Hyogo 657-0013, Japan.

## Abstract

Controlling the temporal waveform of light is the key to a versatile light source in classical and quantum electronics. Although pulse shaping of classical light is mature and has been used in various fields, more advanced applications would be realized by a light source that generates arbitrary quantum light with arbitrary temporal waveforms. We call such a device a quantum arbitrary waveform generator (Q-AWG). The Q-AWG must be able to handle various quantum states of light, which are fragile. Thus, the Q-AWG requires a radically different methodology from classical pulse shaping. Here, we invent an architecture of Q-AWGs that can operate semi-deterministically at a repetition rate over gigahertz in principle. We demonstrate its core technology via generating highly nonclassical states with temporal waveforms that have never been realized before. This result would lead to powerful quantum technologies based on Q-AWGs such as practical optical quantum computing.

## Teaser

A general-purpose quantum light source generates quantum states with temporal waveforms essential for practical quantum computing.

## INTRODUCTION

The development of light sources is the key to innovation in science and technology. An arbitrary waveform generator (AWG), a device that outputs laser light with a desired temporal waveform, is one of the most versatile light source (*1*, *2*). By optimizing the temporal waveform according to purposes, the AWG can solve technical problems in various applications such as coherent control of light-matter interaction (*3*–*5*), ultrafast optical communication (*6*, *7*), spectroscopy (*8*, *9*), and manipulation of quantum entanglement (*10*). The AWG seems to engineer the light flexibly; however, it actually engineers only a very limited degree of freedom from the perspective of quantum optics. This is because an AWG can output only classical states called coherent states among various quantum states of light. Thus, AWGs are not suitable for applications that explicitly use quantum nature of light such as quantum computing (*11*, *12*), quantum networking (*13*–*16*), and quantum sensing (*17*), where a variety of quantum states are required including squeezed states, Fock states, Schrödinger cat states, and so on.

**Fig. 1.**
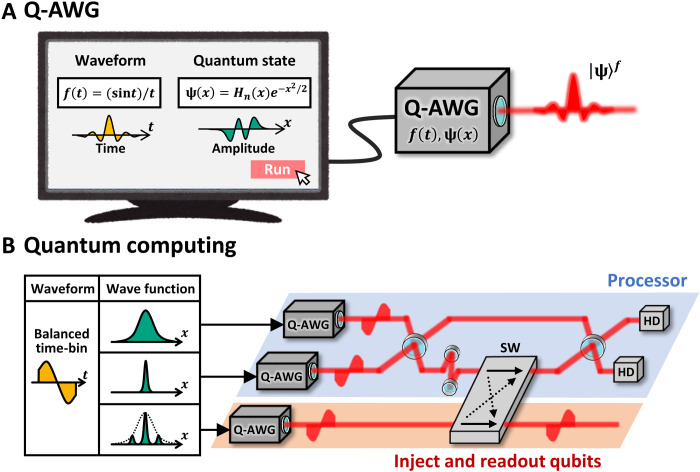
The concept and application of a Q-AWG. (**A**) Operation image. A user specifies the output by a temporal waveform *f* (*t*) and a wave function ψ(*x*). This figure is an example of generating an *n*-photon state with a sinc-shaped temporal waveform. (**B**) Scalable quantum computing using Q-AWGs. Quantum states are efficiently multiplexed in time domain by using a BTB waveform. Qubits that have non-Gaussian wave functions are injected into the processor by an optical switch (SW). Computing is carried out in a measurement-based way using homodyne detectors (HDs). The result of the computing is read out from the processor by the SW.

To get rid of the limitation of an AWG, we propose a concept of a quantum AWG (Q-AWG), a device that outputs an arbitrary quantum state of light with an arbitrary temporal waveform. Figure 1A is an operating image of a Q-AWG. The temporal waveform of the output is specified by a function *f* (*t*) as with an AWG. In a Q-AWG, the quantum state of the output can also be specified by the distribution of optical amplitude. The quantum complex amplitude of light corresponds to an annihilation operator $\hat{a}=(\hat{x}+i\hat{p})/\sqrt{2}$. We can use the probability amplitude of the *x* component, a wave function ψ(*x*), to uniquely specify a quantum state, where ∣ψ(*x*)∣^2^ gives the probability density function of *x*. For example, when we want an *n*-photon state with a sinc-shaped temporal waveform, we set ψ(*x*) ∝ *H_n_*(*x*)*e*^−*x*^2^/2^ and *f*(*t*) ∝ ( sin *t*)/*t* as illustrated in Fig. 1A. Here, *H_n_*(*x*) is the Hermite polynomial. Such a dual degree of freedom of ψ(*x*) and *f* (*t*) makes the Q-AWG distinctly different from the AWG, which only engineers *f* (*t*). Realization of the Q-AWG directly leads to various quantum applications. As an example, Fig. 1B is scalable measurement–based quantum computing that multiplexes quantum states of light in time domain. In this scheme, it is effective to use a balanced time-bin (BTB) waveform (*18*–*22*). By outputting various quantum states with this temporal waveform from Q-AWGs, large-scale, universal, and fault-tolerant optical quantum computing can be performed. The Q-AWG would also play a central role in temporal encoding of quantum information (*23*), real-time quantum measurement (*24*), hyper entanglement (*25*), sensing and mitigation of gravitational effects in satellite-based communication (*26*), and optimal bridging of solid systems such as two-level atoms (*27*), optomechanical oscillators (*28*), and quantum memories (*29*, *30*).

Because Q-AWGs deal with general quantum states, their implementation requires a methodology that is completely different from conventional AWGs. First, Q-AWGs require low-loss generation of output states because quantum states are fragile and easily lose their nonclassical features by optical loss. Therefore, the way of thinking about state preparation in Q-AWGs is much different from that in conventional AWGs, which deal with only loss-tolerant classical states. Second, how to engineer the temporal waveform of non-Gaussian states is unclear. Quantum states are classified into Gaussian and non-Gaussian states depending on whether the wave function ψ(*x*) is Gaussian or non-Gaussian. Non-Gaussian states have especially high nonclassicality and are an essential factor in many quantum applications (*31*–*33*). The method of generating arbitrary Gaussian states, which include coherent states, with an arbitrary temporal waveform is known to some extent (*10*, *18*). Although arbitrary non-Gaussian wave functions can be realized via heralding state generation schemes in principle (*34*), the methodology for realizing arbitrary temporal waveforms has remained elusive. Thus far, waveform engineering of non-Gaussian states has been discussed only in specific heralding schemes. This has resulted in the demonstration of limited types of non-Gaussian states with several simple temporal waveforms in continuous wave (CW) (*24*, *35*–*37*) and pulsed regime (*38*, *39*).

Here, we propose an architecture of Q-AWGs and demonstrate its core technology—generation of an arbitrary non-Gaussian state with an arbitrary waveform. Our idea is to engineer the temporal waveform using a correlation called quantum entanglement. Heralding schemes inherently have quantum entanglement between the output channel and other ancillary channels. The temporal waveform of generated non-Gaussian states is affected by optical filters in the ancillary channels via quantum entanglement. We find that such a filtering realizes arbitrary temporal waveforms in all typical heralding schemes by generating the entanglement from broadband quantum light sources. By incorporating timing control technique (*40*–*42*), high success rate heralding methods (*43*, *44*), and terahertz-bandwidth quantum light sources (*45*, *46*) into the basic idea, the Q-AWG can output states semi-deterministically with a repetition rate over gigahertz. As a demonstration, we generate non-Gaussian states with time-bin (TB) and BTB waveforms, which have never been realized before. This result would lead to practical quantum computing shown in Fig. 1B and to realization of the Q-AWG—an ultimate quantum light source.

## RESULTS

### Temporal waveform and wave function of quantum states

We take a rotating frame so that the career frequency becomes ω = 0. The amplitude of classical light with a temporal waveform *f* (*t*) is given by *f*(*t*)*a_c_*, where *a_c_* is a complex number. A quantum counterpart of this amplitude is a creation operator given by

$$\hat{a}[f]≔\int f(t)\hat{a}(t)\;\mathrm{dt}$$ (1)

where $\hat{a}(t)$ is the instantaneous annihilation operator satisfying a commutation relation $\left[\hat{a}(t),{\hat{a}}^{†}(t^{\prime })]=\delta (t-t^{\prime }\right)$. In the following, we suppose that ∫∣*f*(*t*)∣^2^ *dt* = 1 for that $\hat{a}[f]$ satisfies $[\hat{a}[f],{\hat{a}}^{†}[f]]=1$. Because $\hat{a}[f]$ is not an operator of an observable, operators of photon number $\hat{n}[f]≔{\hat{a}}^{†}[f]\hat{a}[f]$ or real and imaginary components of the complex amplitude $\hat{x}[f]≔\frac{\hat{a}[f]+{\hat{a}}^{†}[f]}{\sqrt{2}},\hat{p}[f]≔\frac{\hat{a}[f]-{\hat{a}}^{†}[f]}{\sqrt{2}i}$ are often used to describe quantum states. A quantum state with a temporal waveform *f* (*t*) is given by

$${|\psi \rangle }^{f}=\sum_{n=0}^{\infty }\psi_{n}{|n\rangle }^{f}=\int \psi (x){|x\rangle }^{f}\mathrm{dx}$$ (2)

where $\hat{n}[f]{|n\rangle }^{f}=n{|n\rangle }^{f}$ and $\hat{x}[f]{|x\rangle }^{f}=x{|x\rangle }^{f}$. The goal of a Q-AWG is to generate a quantum state ∣ψ〉*^f^* that has arbitrary temporal waveform *f* (*t*) and wave function ψ(*x*).

### Architecture and operating principle

**Fig. 2.**
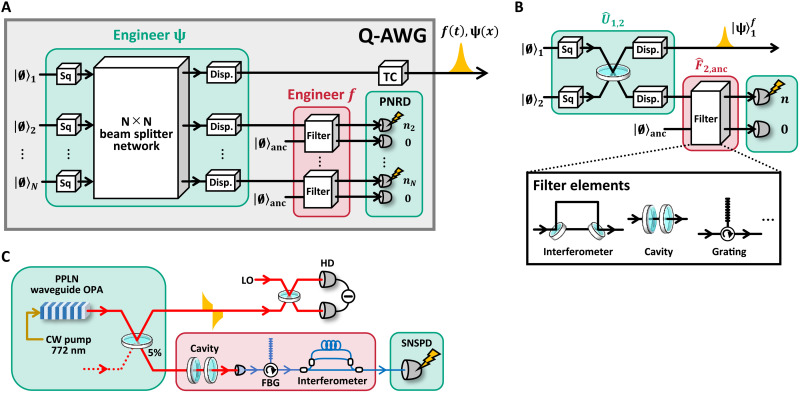
The diagram of the proposed Q-AWG and setup of the experiment. (**A**) The architecture of the Q-AWG. Arbitrary non-Gaussian states with arbitrary temporal waveforms are generated by a heralding scheme. A timing controller (TC) adjusts the emission timing of the state. CW, continuous wave; PNRD, photon number resolving detector. (**B**) The detail of waveform engineering in a two-channel case. The experiment we conduct is the two-channel case with the timing controller omitted. (**C**) Experimental setup. PPLN, periodically poled LiNbO_3_; OPA, optical parametric amplifier; LO, local oscillator; FBG, fiber Bragg grating; SNSPD, superconducting nanostrip photon detector.

First, we introduce an architecture of the Q-AWG shown in Fig. 2A. The Q-AWG generates a non-Gaussian state via the heralding scheme, where photon number measurement is performed on a subsystem of a Gaussian state defined on multiple channels (*34*, *43*). Arbitrary Gaussian states can be generated deterministically by using squeezing, beam splitter, and displacement operations. In the heralding scheme, there exists quantum entanglement between the output channel and photon detection channels, and photon detection affects the generated state on the output channel via the entanglement. In principle, we can realize arbitrarily wave function ψ(*x*) of the generated state by changing the initial Gaussian state and the number of photons detected (*34*). In the heralding scheme, optical filters inserted on the photon detection channels can affect the temporal waveform of the generated state via entanglement. Supposing the initial Gaussian state is broadband CW quantum light, we can realize an arbitrary temporal waveform *f* (*t*) of the generated state by inserting passive linear filters with an impulse response *g*(*t*) ∝ *f*(−*t*) before the photon detectors. Note that there are no filters placed on the output channel. In the AWG, filters or modulators, which are usually lossy, directly engineer the temporal waveform of the output classical light. In the Q-AWG, on the other hand, all these elements can be pushed into the photon detection channels. In this situation, the heralding method using weakly squeezed inputs, which is widely used at present, can generate high-purity non-Gaussian states regardless of the loss of the photon detection channel.

Because heralding succeeds only when a specific result is obtained in the photon number measurements, the desired state is generated at random timing. A Q-AWG can work semi-deterministically, however, by introducing timing controllers that adjust the emission timing of the output states by giving a time delay τ. The examples of timing controllers are an optical delay line with continuously tunable τ (*40*) and quantum memories based on optical loop circuits, which can realize discrete but relatively large τ (*41*, *42*). One advantage of our method is that we suppose a general Gaussian initial state. Thanks to this, our method can be applied to recently developed efficient heralding methods (*43*, *44*) with a success probability around 10^−3^ to 10^−2^. By incorporating these efficient heralding methods and terahertz-bandwidth quantum light sources (*45*, *46*) into our method, where the bandwidth roughly corresponds to the trial rate of state generation, the Q-AWG in Fig. 2A can operate semi-deterministically at a repetition rate over gigahertz.

Next, we introduce the working principle of the Q-AWG in a two-channel case shown in Fig. 2B. We suppose that CW-squeezed light in channels 1 and 2 interferes at a beam splitter, and each channel is displaced. We express this initial Gaussian state as Uˆ1,2∣0〉1∣0〉2∣0〉anc, where ∣0〉j is a multimode vacuum state of channel *j* satisfying aˆj(t)∣0〉j=0 for all *t*. A passive filter consisting of linear optics, which has an ancillary channel in quantum description (*47*), is inserted in channel 2. We perform photon number measurement on the filter outputs. When we detect *n* photons on channel 2 at *t* = 0 and detect no photon on the ancillary channel, the heralded state on channel 1 is given by

∣Ψ1〉∝〈0∣anc〈0∣2aˆ2(t=0)nFˆ2,ancUˆ1,2∣0〉1∣0〉2∣0〉anc (3)

where ${\hat{F}}_{2,\text{anc}}$ is a filter operator.

Suppose we aim to realize a temporal waveform *f* (*t*). Generally, *f* (*t*) is a complex function, but for the time being, we suppose *f* (*t*) is a real function. We introduce the following assumptions: (i) Squeezing and beam splitter operation are broadband and frequency independent within the spectrum of *f* (*t*), and (ii) the filter transmittance about channel 2 is characterized by an impulse response *g*(*t*) ∝ *f*(−*t*) ∈ ℝ. The assumption on the beam splitter is usually satisfied in experiments, and thus, the assumptions on the squeezed light and the filter are the key. Under these assumptions, ∣Ψ〉_1_ in Eq. 3 can be decomposed to a direct product of a single-mode state about *f* (*t*) and a multimode state with orthogonal waveforms to *f* (*t*) as follows

$${|\Psi \rangle }_{1}={|\psi \rangle }_{1}^{f}⊗{|\phi \rangle }_{1}^{\left\{f_{⊥}\right\}}$$ (4)

$${|\psi \rangle }_{1}^{f}\propto {\;}_{2}^{f}{\langle n|G\rangle }_{1,2}^{f}$$ (5)

Here, ${|G\rangle }_{1,2}^{f}$ is an arbitrary Gaussian state with a temporal waveform *f* (*t*). See Materials and Methods for the derivation. ${|\psi \rangle }_{1}^{f}$ is a pure non-Gaussian state due to the effect of photon number measurement except for the cases where ${|G\rangle }_{1,2}^{f}$ is a separable state or *n* = 0. Note that Eq. 5 is equivalent to the generalized heralding scheme supposing an arbitrary two-channel Gaussian input (*43*, *44*). Because we can implement a filter with an arbitrary impulse response *g*(*t*) ∈ ℂ (*39*, *48*), our method can tailor arbitrary temporal waveforms given by a real function. In addition, it is always possible to maximize the generation rate of non-Gaussian states by constructing a 100% efficiency filter that transmits the desired spectrum only to one output channel (*48*). Existence of such an efficient filter is important because low generation rate is often problematic in heralding schemes.

In Eq. 4, a Gaussian state ${|\phi \rangle }_{1}^{\left\{f_{⊥}\right\}}$ is generated with waveforms orthogonal to the target waveform *f* (*t*). In principle, the target state ${|\psi \rangle }_{1}^{f}$ can be separated from ${|\phi \rangle }_{1}^{\left\{f_{⊥}\right\}}$ by a filter. Let us suppose injecting the state ${|\psi \rangle }_{1}^{f}⊗{|\phi \rangle }_{1}^{\left\{f_{⊥}\right\}}$ and an ancillary vacuum state ${|\tilde{0}\rangle }_{2}$ to a two-input–two-output filter that transmits *f* (t) component with 100% efficiency. The outputs of the filter are given by ${|\psi \rangle }_{1}^{f}⊗{|\tilde{0}\rangle }_{1}^{\left\{f_{⊥}\right\}}$ and ${|\tilde{0}\rangle }_{2}^{f}⊗{|\phi \rangle }_{2}^{\left\{f_{⊥}\right\}}$. This filtering of the generated non-Gaussian states is usually much more loss sensitive than the filtering in the photon measurement channels, and thus, obtaining the state ${|\psi \rangle }_{1}^{f}⊗{|\tilde{0}\rangle }_{1}^{\left\{f_{⊥}\right\}}$ is not very easy. In reality, however, this separation will hardly be necessary. In quantum applications, we often perform amplitude measurement (*12*, *16*) or photon detection (*11*, *13*) of the state with a temporal waveform *f* (*t*). Let us suppose that we want to measure the value $\hat{x}[f]≔\int f(t)\hat{x}(t)\;\mathrm{dt}$ in the amplitude measurement, where $\hat{x}(t)≔\frac{\hat{a}(t)+{\hat{a}}^{†}(t)}{\sqrt{2}}$. In this situation, the existence of ${|\phi \rangle }_{1}^{\left\{f_{⊥}\right\}}$ does not matter because we can extract the value of *x*[*f*] from the result of CW homodyne measurement *x*(*t*) by using a function *f* (*t*). On the other hand, photon measurement requires ${|\phi \rangle }_{1}^{\left\{f_{⊥}\right\}}={|\tilde{0}\rangle }_{1}^{\left\{f_{⊥}\right\}}$, so that photons of ${|\phi \rangle }_{1}^{\left\{f_{⊥}\right\}}$ do not contaminate the measurement results. By supposing the input state to be an Einstein-Podolsky-Rosen state, we get ${|\psi \rangle }_{1}^{f}⊗{|\phi \rangle }_{1}^{\left\{f_{⊥}\right\}}={|n\rangle }_{1}^{f}⊗{|\tilde{0}\rangle }_{1}^{\left\{f_{⊥}\right\}}$. Because applications using photon measurement mostly make use of Fock states ${|n\rangle }_{1}^{f}$ (*11*, *13*), the Q-AWG can generate desired states without state separation by a filter.

More general quantum state ${|\psi \rangle }_{1}^{f}$ can be generated using *N* channels. Supposing we detect *n_j_* photons in channel *j* (2 ≤ *j* ≤ *N*) after the filters with *g*(*t*) ∝ *f*(−*t*) ∈ ℝ, we can generate a state given by

∣ψ〉1f∝ Nf〈nN∣⋯〈n3∣3f〈n2∣G〉2f1,2,⋯,Nf (6)

where ${|G\rangle }_{1,2,\cdots ,N}^{f}$ is an arbitrary Gaussian state with a temporal waveform *f* (*t*) on channel 1 to *N*. One example of *N*-channel heralding uses Einstein-Podolsky-Rosen state, where one mode of the state is divided into *N* – 1 channels and displaced before photon number measurement. By supposing *n_j_* = 1 (2 ≤ *j* ≤ *N*), ${|\psi \rangle }_{1}^{f}$ can be an arbitrary superposition up to *N* – 1 photons (*34*). Thus, arbitrary single-mode state can be generated when *N* → ∞. Even so, discussing the case of general ${|G\rangle }_{1,2,\cdots ,N}^{f}$ is important because many useful states can be generated much more efficiently in this situation (*43*, *44*).

Last, we show two possible ways to realize a temporal waveform given by a complex function. One is to add a phase *e*^*i*θ(*t*)^ to the generated state; the temporal waveform is converted from *f* (*t*) to *f*(*t*)*e*^*i*θ(*t*)^. Another way is to use an Einstein-Podolsky-Rosen state as an input; in this case, we can realize an arbitrary complex waveform *f* (*t*) by using a filter with an impulse response *g*(*t*) ∝ *f*(−*t*) ∈ ℂ. See Materials and Methods for the detail.

### Experiment

As a demonstration, we generate Schrödinger cat states in TB and BTB waveforms. The cat states are non-Gaussian states defined as ∣α〉 ± ∣−α〉, where ∣α〉 is a coherent state with amplitude α. The target waveforms are given by growth rate Γ and bin duration Δ*t* as follows

$$f_{\text{TB}}(t)=\sqrt{\frac{2\Gamma }{\text{exp}\;[2\Gamma \Delta t]-1}}\times \{\begin{array}{cc} 0 & \left(t<0,\Delta t\leq t\right)\\ \text{exp}\;[\Gamma t] & \left(t\geq 0\right) \end{array}$$ (7)

$$f_{\text{BTB}}(t)=\sqrt{\frac{\Gamma }{\text{exp}\;[2\Gamma \Delta t]-1}}\times \{\begin{array}{cc} 0 & \left(t<0,2\Delta t\leq t\right)\\ \text{exp}\;[\Gamma t] & \left(0\leq t<\Delta t\right)\\ -\text{exp}\;[\Gamma (t-\Delta t)] & \left(\Delta t\leq t<2\Delta t\right) \end{array}$$ (8)

where TB and BTB denote TB and BTB, respectively. These functions have nonzero values only at finite time domain, and the BTB waveform has no career component because ∫*f*_BTB_(*t*) *dt* = 0. The BTB waveform is suitable for time-domain multiplexing of quantum states and thus intensively adopted as a computational basis in scalable optical quantum computers (*18*–*22*). Thus far, those waveforms have not been demonstrated in non-Gaussian state generation.

We conduct photon subtraction (*49*) to generate the cat states by the setup shown in Fig. 2C. We demonstrate the case of *n* = 1 and thus expect that the cat states ∣α〉 −∣−α〉 with ∣α∣ ∼ 1 are generated. In our method, a broadband CW squeezer and a filter with a desired impulse response are the key. As a broadband source, a single-pass waveguide optical parametric amplifier (OPA) is a promising candidate. In this experiment, we use the low-loss periodically poled LiNbO_3_ waveguide OPA module (*45*) we have recently developed. Our module can generate terahertz -bandwidth CW-squeezed light at the wavelength of 1545 nm with high purity sufficient for non-Gaussian state generation (*46*). The squeezed light is emitted into free space, and then 5% of it is tapped for the photon detection. We construct two filters with impulse response *g*_TB_(*t*) ∝ *f*_TB_(−*t*) and *g*_BTB_(*t*) ∝ *f*_BTB_(−*t*) before the photon detector. On the basis of the optical quantum information processors reported so far (*20*, *22*), we set the parameter in Eqs. 7 and 8 as Δ*t* = 20 ns and Γ = 2π × 8.2 MHz. The filters consist of Mach-Zehnder interferometers, optical cavities, and fiber Gragg gratings. These elements are suitable to realize filters with various bandwidths from megahertz order to terahertz order, and we can approximate arbitrary impulse response with arbitrary accuracy using those elements (*48*). We detect photons with a superconducting nanostrip photon detector (SNSPD) (*50*), and the timing jitter of photon detection is about 100 ps, sufficiently small for this experiment. Each click at the SNSPD heralds a cat state in the other channel. We omit the photon number measurement on the ancillary outputs of the filter because simultaneous photon detection at the outputs is rare.

### Evaluation

We evaluate the filters and the generated states by using homodyne detectors with 200-MHz bandwidth. Supposing θ_LO_ is the phase of the CW local oscillator (LO) beam, we measure the instantaneous amplitude ${\hat{x}}_{\theta_{\text{LO}}}(t)≔[\hat{a}(t)e^{-i\theta_{\text{LO}}}+{\hat{a}}^{†}(t)e^{i\theta_{\text{LO}}}]/\sqrt{2}=\hat{x}(t)\text{cos}\;\theta_{\text{LO}}+\hat{p}(t)\text{sin}\;\theta_{\text{LO}}$. Because ${\hat{x}}_{\theta_{\text{LO}}}[f]≔\hat{x}[f]\text{cos}\;\theta_{\text{LO}}+\hat{p}[f]\text{sin}\;\theta_{\text{LO}}=\int f(t){\hat{x}}_{\theta_{\text{LO}}}(t)\;\mathrm{dt}$, we can extract the information of the target states using the measurement result ${\hat{x}}_{\theta_{\text{LO}}}(t)$ and the waveform *f* (*t*).

First, we evaluate the impulse response of the filters. We input weak pulsed-laser light to the filters and measure the output for 1000 times with random θ_LO_. The duration of the input pulse is less than 1 ps. We estimate the impulse response by applying principal components analysis (*51*) to the autocorrelation of homodyne signal. The waveform of the generated states is also estimated by principal components analysis, where we measure 20,000 events for each phase θ_LO_ = 0,30° ,60° ,90° ,120°, and 150°. We measure vacuum states in the same condition as a reference for the shot-noise level. The efficiency of the homodyne detector for generated state evaluation is 0.93. Experimentally generated states are always mixed states, and thus, Wigner functions are often used for evaluation rather than wave functions. In particular, the mixed state is usually regarded as non-Gaussian state only when its Wigner function has negative values. Using the estimated waveform *f* (*t*), we calculate the amplitude ${\hat{x}}_{\theta_{\text{LO}}}[f]$ and reconstruct the Wigner function of the generated states by quantum state tomography (*52*).

**Fig. 3.**
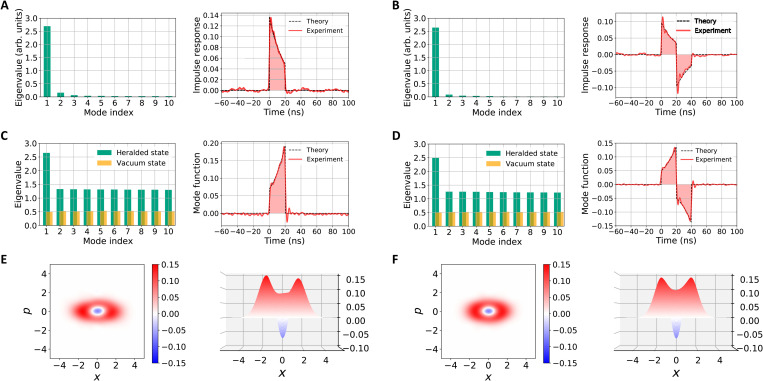
Results of the experiment. (**A**) Results of principal components analysis about the filter for the TB waveform. The bar chart is the eigenvalues, and the line chart is the first eigenfunction. (**B**) Similar figures to (A) about the BTB waveform. (**C**) Results of principal components analysis about the heralding generation of states in the TB waveform. The eigenvalues are the case of θ_LO_ = 0°, and the waveform is estimated from all the measured data. (**D**) Similar figures to (C) about the BTB waveform. (**E**) Wigner function of the state generated in the estimated waveform shown in (C). (**F**) Wigner function of the state generated in the estimated waveform shown in (D).

Figure 3 shows the experimental results. Figure 3 (A and B) is the result of impulse response evaluation about TB and BTB waveforms, respectively. These figures show the eigenvalues and the first eigenfunction of principal components analysis. In both figures, the first eigenvalue is much larger than the other eigenvalues, indicating that the impulse response of this filter can be expressed as a real function and that the cavity and interferometer of the filter are properly controlled. The first eigenfunction is the estimated impulse response of the filter. We can observe finite time duration of the impulse response as expected. The mode match with the theoretical prediction is 0.971 for the TB waveform and 0.954 for BTB, and thus, the filters are implemented with a good accuracy. Figure 3 (C and D) is the result of waveform evaluation of the generated quantum states. The green bars in these figures are the eigenvalues of principal components analysis of the generated states with θ_LO_ = 0°. Corresponding eigenfunctions are the waveforms of those components. We can see that only the first eigenvalue is outstanding, indicating that single-mode non-Gaussian states are generated with the waveform given by the first eigenfunction. The orange bars are the variance of the amplitude of vacuum states with those waveforms. We estimate the waveforms of the generated states as the average of the first eigenfunctions of all θ_LO_. The line charts in Fig. 3 (C and D) are the estimated waveforms. Because the squeezed light is sufficiently broadband, these functions are given by the time-reversed impulse responses shown in Fig. 3 (A and B). The mode match with the theoretical prediction is 0.958 for the TB waveform and 0.954 for BTB. Figure 3 (E and F) is the Wigner functions of the states in the estimated TB and BTB waveforms. These states are non-Gaussian states with high nonclassicality because each Wigner function has negative value of −0.070 ± 0.002 and −0.068 ± 0.002 at the origin of the phase space, respectively. These states best approximate a cat state with ∣α∣ = 0.94, where the fidelities are *F* = 0.564 ± 0.001 for the TB waveform and *F* = 0.604 ± 0.03 for the BTB. The fidelity can get much better by improving the main imperfections of the system: loss at the waveguide OPA, inefficiency of the homodyne detector and SNSPD, and fake count of the SNSPD. From all the above, we have successfully demonstrated the core technology of the proposed Q-AWG via generating non-Gaussian states in the TB and BTB waveforms.

## DISCUSSION

Our achievement is twofold: First, we have proposed the concept of the Q-AWG and its architecture based on heralding schemes using CW; second, we have demonstrated the core technology of the Q-AWG, arbitrary control of the temporal waveform of non-Gaussian states, via realizing waveforms essential for practical optical quantum computing (*18*–*22*).

As a future prospect, generating of quantum states with wider bandwidth is a straightforward task. Widening the bandwidth has the following two implications. First, the wider the bandwidth, the shorter the delay lines in a filter and a timing controller, and the more compact the whole system will be. Therefore, it becomes easy to realize a complex temporal waveform by a filter consists of many elements, to make the Q-AWG semi-deterministic, and to integrate the system on a chip by the recent waveguide technology (*53*). Another implication is improvement of the state generation rate. This is because the bandwidth of the generated states, that is, the bandwidth of the filter, is roughly regarded as the trial rate of state generation in a heralding scheme. As fast detection of amplitude and photon number are having been developed (*45*, *54*, *55*), generation of non-Gaussian states with gigahertz bandwidth will be possible before long. If related technologies develop further and the terahertz bandwidth of the waveguide OPA is fully exploited, then a semi-deterministic Q-AWG with a gigahertz-order repetition rate can be realized.

Multimode Q-AWGs are also an interesting research direction. By using filters with different impulse responses in a *N*-channel heralding scheme, our method can be generalized to arbitrary control of multimode non-Gaussian states. Multimode Q-AWGs would be used to increase the capacity of quantum information processing by multiplexing quantum states in frequency domain (*56*) or to generate logical states for fault-tolerant quantum computing (*23*).

Although our experimental result is directly relevant to optical quantum computing, the Q-AWG can also be applied to other applications such as quantum networking (*27*–*30*) and quantum sensing (*26*). In the future, it is quite possible that useful quantum light or powerful applications that are not yet known will be proposed. We can flexibly meet any such requirements because our method allows arbitrary control of quantum light at any wavelength. The concept of the Q-AWG and our technical achievements would be a key of optical quantum technology.

## MATERIALS AND METHODS

### Decomposition of CW multimode states

We show the derivation of Eqs. 4 and 5. A fundamental operator of wave packet mode *f* is a creation operator given by

$$\hat{a}[f]=\int f(t)\hat{a}(t)\;\mathrm{dt}=\int \tilde{f}(-\omega )\hat{\tilde{a}}(\omega )\;d\omega$$ (9)

where $\hat{a}(t)$ and $\hat{\tilde{a}}(\omega )$ are the instantaneous and monochromatic annihilation operators satisfying commutation relations $\left[\hat{a}(t),{\hat{a}}^{†}(t^{\prime })]=\delta (t-t^{\prime }),[\hat{\tilde{a}}(\omega ),{\hat{\tilde{a}}}^{†}(\omega^{\prime })]=\delta (\omega -\omega^{\prime }\right)$. These operators are transformed to each other by $\hat{a}(t)=\frac{1}{\sqrt{2\pi }}\int \hat{\tilde{a}}(\omega )e^{-i\omega t}\;d\omega$ and $\hat{\tilde{a}}(\omega )=\frac{1}{\sqrt{2\pi }}\int \hat{a}(t)e^{i\omega t}\;\mathrm{dt}$. The mode functions $f(t),\tilde{f}(\omega )$ satisfy

$$\int {|f(t)|}^{2}\;\mathrm{dt}=1\;,\;\int {|\tilde{f}(\omega )|}^{2}\;d\omega =1$$ (10)

$$\tilde{f}(\omega )=\frac{1}{\sqrt{2\pi }}\int f(t)e^{i\omega t}\;\mathrm{dt}\;,\;\;f(t)=\frac{1}{\sqrt{2\pi }}\int \tilde{f}(\omega )e^{-i\omega t}\;d\omega$$ (11)

Note that the mode function *f* (*t*) is referred as a temporal waveform in the main text. Equation 10 guarantees $[\hat{a}[f],{\hat{a}}^{†}[f]]=1$. The Fock states ${\left\{|n_{f}\rangle \right\}}_{n=0}^{\infty }$ defined as the eigenstates of ${\hat{a}}^{†}[f]\hat{a}[f]$ are a complete orthonormal system of the mode *f*.

A quantum filter requires at least one ancillary input (*47*). We explicitly deal with two-mode filters, but the same argument holds for the multimode case. A passive filter operator on a channel *j*, which we denote ${\hat{F}}_{j,\text{anc}}$, gives a transformation

$${\hat{F}}_{j,\text{anc}}^{†}{\hat{a}}_{j}(t){\hat{F}}_{j,\text{anc}}=\int [g(\tau ){\hat{a}}_{j}(t-\tau )-\{\delta (\tau )-g(\tau )\}{\hat{a}}_{\text{anc}}(t-\tau )]\;d\tau$$ (12)

where *g*(*t*) is the impulse response of the filter. Then, Eq. 3 is given by

∣Ψ〉1∝ anc〈0∣〈0∣2aˆ2(t=0)nFˆ2,ancUˆ1,2∣0〉1∣0〉2∣0〉anc= 〈0∣anc〈0∣2[∫[g(τ)aˆj(−τ)−{δ(τ)−g(τ)}aˆanc(−τ)] dτ]nUˆ1,2∣0〉1∣0〉2∣0〉anc∝ 〈0∣2aˆ2[N(gR)]nUˆ1,2∣0〉1∣0〉2 (13)

where we use Fˆ2,anc∣0〉2∣0〉anc=∣0〉2∣0〉anc and *N*( · ) denotes normalization. The Gaussian operator ${\hat{U}}_{1,2}$ is given by

$${\hat{U}}_{1,2}={\hat{D}}_{1}(\alpha_{1},\omega_{d}){\hat{D}}_{2}(\alpha_{2},\omega_{d}){\hat{B}}_{1,2}{\hat{S}}_{1}[r_{1}]{\hat{S}}_{2}[r_{2}]$$ (14)

where CW squeezing operator ${\hat{S}}_{j}[r]$, frequency-independent beam splitter operator ${\hat{B}}_{j,k}(\kappa ,\nu ,\mu )$, and monochromatic displacement operator ${\hat{D}}_{j}(\alpha ,\omega_{d})$ are defined as

$${\hat{S}}_{j}[r]=\text{exp}\;[\frac{1}{2}({\hat{P}}_{j}^{†}[r]-{\hat{P}}_{j}[r])]$$ (15)

$$\;{\hat{P}}_{j}^{†}[r]=\iint r(t_{1}-t_{2}){\hat{a}}_{j}^{†}(t_{1}){\hat{a}}_{j}^{†}(t_{2})\;{\mathrm{dt}}_{1}{\mathrm{dt}}_{2}=\int \tilde{r}(\omega ){\tilde{a}}_{j}^{†}(\omega ){\tilde{a}}_{j}^{†}(-\omega )\;d\omega$$ (16)

$${\hat{B}}_{j,k}(\kappa ,\nu ,\mu )=\text{exp}\;[i\frac{\nu }{2}\hat{L}]\cdot \text{exp}\;[\frac{\kappa }{2}\hat{M}]\cdot \text{exp}\;[i\frac{\mu }{2}\hat{L}]$$ (17)

$$\begin{array}{c} \hat{L}=\int [{\tilde{a}}_{j}^{†}(\omega ){\tilde{a}}_{j}(\omega )-{\tilde{a}}_{k}^{†}(\omega ){\tilde{a}}_{k}(\omega )]d\omega ,\hat{M}\\ =\int [{\tilde{a}}_{j}^{†}(\omega ){\tilde{a}}_{k}(\omega )-{\tilde{a}}_{k}^{†}(\omega ){\tilde{a}}_{j}(\omega )]d\omega \end{array}$$ (18)

$${\hat{D}}_{j}(\alpha ,\omega_{d})=\text{exp}\;[\alpha {\tilde{a}}_{j}^{†}(\omega_{d})-\alpha^{*}{\tilde{a}}_{j}(\omega_{d})]$$ (19)

From the time-reversal symmetry *r*(*t*) = *r*(−*t*), $\tilde{r}(\omega )$ satisfies $\tilde{r}(\omega )=\tilde{r}(-\omega )$.

In the following, we consider mode decomposition of the operators ${\hat{B}}_{j,k}(\kappa ,\nu ,\mu )$, ${\hat{D}}_{j}(\alpha ,\omega_{d})$, and ${\hat{S}}_{j}[r_{j}]$. The idea of mode decomposition in quantum optical engineering was first introduced in (*57*). For arbitrary wave packet mode *f*, the operators ${\hat{B}}_{j,k}(\kappa ,\nu ,\mu )$ and ${\hat{D}}_{j}(\alpha ,\omega_{d})$ can be decomposed to direct products of operators about mode *f* and orthogonal modes. We can always define a complete orthonormal system ${\left\{{\tilde{f}}_{\left(l\right)}(\omega )\right\}}_{l=1}^{\infty }$, where ${\tilde{f}}_{\left(1\right)}(\omega )=\tilde{f}(\omega )$. From the property of completeness $\sum_{l=1}^{\infty }{\tilde{f}}_{\left(l\right)}(\omega ){\tilde{f}}_{\left(l\right)}^{*}(\omega \prime )=\delta (\omega -\omega \prime )$, we can derive

$$\begin{array}{cc} \sum_{l=1}^{\infty }{\hat{a}}_{j}^{†}[f_{\left(l\right)}]{\hat{a}}_{k}[f_{\left(l\right)}] & =\sum_{l=1}^{\infty }\int {\tilde{f}}_{\left(l\right)}^{*}(-\omega ){\tilde{a}}_{j}^{†}(\omega )\;d\omega \cdot \int {\tilde{f}}_{\left(l\right)}(-\omega \prime ){\tilde{a}}_{k}(\omega \prime )\;d\omega \prime \\ & =\iint {\tilde{a}}_{j}^{†}(\omega ){\tilde{a}}_{k}(\omega \prime )\sum_{l=1}^{\infty }{\tilde{f}}_{\left(l\right)}^{*}(-\omega ){\tilde{f}}_{\left(l\right)}(-\omega \prime )\;d\omega d\omega \prime \\ & =\int {\tilde{a}}_{j}^{†}(\omega ){\tilde{a}}_{k}(\omega )\;d\omega \end{array}$$ (20)

Then, the rotation term of ${\hat{B}}_{j,k}(\kappa ,\nu ,\mu )$ can be decomposed as follows

$$\begin{array}{cc} \text{exp}\;[i\frac{\nu }{2}\hat{L}] & =\text{exp}\;[i\frac{\nu }{2}\int [{\hat{a}}_{j}^{†}(\omega ){\hat{a}}_{j}(\omega )-{\hat{a}}_{k}^{†}(\omega ){\hat{a}}_{k}(\omega )]d\omega ]\\ & =\text{exp}\;[i\frac{\nu }{2}\sum_{l=1}^{\infty }[{\hat{a}}_{j}^{†}[f_{\left(l\right)}]{\hat{a}}_{j}[f_{\left(l\right)}]-{\hat{a}}_{k}^{†}[f_{\left(l\right)}]{\hat{a}}_{k}[f_{\left(l\right)}]]]\\ & ={⊗}_{l=1}^{\infty }\text{exp}\;[i\frac{\nu }{2}[{\hat{a}}_{j}^{†}[f_{\left(l\right)}]{\hat{a}}_{j}[f_{\left(l\right)}]-{\hat{a}}_{k}^{†}[f_{\left(l\right)}]{\hat{a}}_{k}[f_{\left(l\right)}]]] \end{array}$$ (21)

The term $\text{exp}\;[i\frac{\kappa }{2}\hat{M}]$ is also decomposed in the same way. Therefore, ${\hat{B}}_{j,k}(\kappa ,\nu ,\mu )$ can be decomposed to a direct product of beam splitter operations on modes ${\left\{f_{\left(l\right)}\right\}}_{l=1}^{\infty }$. As for ${\hat{D}}_{j}(\alpha ,\omega_{d})$, we use

$$\begin{array}{c} \sum_{l=1}^{\infty }{\tilde{f}}_{\left(l\right)}(-\omega_{d}){\hat{a}}_{j}^{†}[f_{\left(l\right)}]=\int {\tilde{a}}^{†}(\omega )\sum_{l=1}^{\infty }{\tilde{f}}_{\left(l\right)}(-\omega_{d}){\tilde{f}}_{\left(l\right)}^{*}(-\omega )\;d\omega \\ ={\tilde{a}}^{†}(\omega_{d}) \end{array}$$ (22)

From this relation, ${\hat{D}}_{j}(\alpha ,\omega_{d})$ is decomposed as follows

$$\begin{array}{cc} {\hat{D}}_{j}(\alpha ,\omega_{d}) & =\text{exp}\;[\alpha {\tilde{a}}_{j}^{†}(\omega_{d})-\alpha^{*}{\tilde{a}}_{j}(\omega_{d})]\\ & ={⊗}_{l=1}^{\infty }\text{exp}\;[({\tilde{f}}_{\left(l\right)}(-\omega_{d})\alpha ){\hat{a}}_{j}^{†}[f_{\left(l\right)}]-({\tilde{f}}_{\left(l\right)}(-\omega_{d})\alpha )*{\hat{a}}_{j}[f_{\left(l\right)}]] \end{array}$$ (23)

This equation means ${\hat{D}}_{j}(\alpha ,\omega_{d})$ displaces each mode *f*_(*l*)_ by the amount of ${\tilde{f}}_{\left(l\right)}(-\omega_{d})\alpha$.

Yoshikawa *et al.* (*58*) shows that for all *f*, we can find an orthogonal mode function $f_{⊥}(t)$ and decompose the photon-pair creation operator ${\hat{P}}_{j}^{†}[r]$ accordingly

$$\begin{array}{c} {\hat{P}}_{j}^{†}[r]=\;||f^{*}r_{j}||(\sqrt{M[f,r_{j}]}\;{\hat{a}}_{j}^{†}{\left[f\right]}^{2}+2\sqrt{1-M[f,r_{j}]}\;{\hat{a}}_{j}^{†}[f]{\hat{a}}_{j}^{†}[f_{⊥}])+\\ \left(\text{irrelevant terms}\right) \end{array}$$ (24)

where *M*[*f*, *r_J_*] = ∣〈f^*^, *N*(f^*^r_j_)〉∣^2^. The irrelevant terms commute with ${\hat{a}}_{j}[f]$. Equation 24 shows that the operator ${\hat{S}}_{j}[r_{j}]$ can be decomposed to a direct product about mode *f* and irrelevant modes when *M*[*f*, *r_j_*] = 1. This condition is satisfied when *f* (*t*) is a real function, and *r_j_*(*t*) can be regarded as a δ function, that is, the squeezing operation has much broader bandwidth compared to the bandwidth of the filter in the measurement channel. From the assumptions introduced in Results, Eq. 3 is given by

∣Ψ〉1∝ 〈0∣2aˆ2[f]n Uˆ1,2[f]⊗Uˆ1,2[{f⊥}]∣0〉1∣0〉2= 〈n∣2fUˆ1,2[f]∣0〉1f∣0〉2f⊗〈0∣2{f⊥}Uˆ1,2[{f⊥}]∣0〉1{f⊥}∣0〉2{f⊥} (25)

where ${\hat{U}}_{1,2}[f]$ and ${\hat{U}}_{1,2}[\{f_{⊥}\}]$ are Gaussian operators on mode *f* and all modes orthogonal to *f*, respectively. ${\hat{U}}_{1,2}[f]$ consists of squeezing, beam splitter, and displacement operators, and thus, the state ${\hat{U}}_{1,2}[f]{|0\rangle }_{1}^{f}{|0\rangle }_{2}^{f}$ can be an arbitrary Gaussian state by choosing proper parameters *r*_1_, *r*_2_, κ, ν, μ, ω*_d_*, α_1_, and α_2_. Therefore, Eq. 3 is given by

∣Ψ〉1∝ 〈n∣G〉2f1,2f⊗∣χ〉1{f⊥} (26)

which is equivalent to Eqs. 4 and 5.

### Realization of complex mode function

Let us consider a special case Uˆ1,2∣0〉1∣0〉2=Dˆ1(α1,ωd)Dˆ2(α2,ωd)Tˆ1,2[r]∣0〉1∣0〉2, where the two-mode squeezing operator ${\hat{T}}_{1,2}[r]$ is given by

$$\;{\hat{T}}_{1,2}[r]=\text{exp}\;({\hat{Q}}^{†}[r]-\hat{Q}[r])$$ (27)

$${\hat{Q}}^{†}[r]=\int \tilde{r}(\omega ){\tilde{a}}_{1}^{†}(\omega ){\tilde{a}}_{2}^{†}(-\omega )\;d\omega$$ (28)

The two-mode squeezed state Tˆ1,2[r]∣0〉1∣0〉2 is also known as an Einstein-Podolsky-Rosen state. We suppose *g*(*t*) ∝ *f*(−*t*) ∈ ℂ. When $\tilde{r}(\omega )$ is sufficiently broadband than $\tilde{g}(\omega )$, we get the following equation as we prove shortly after

Uˆ1,2∣0〉1∣0〉2=Uˆ1,2′[f,f*]∣0〉1f∣0〉2f*⊗Uˆ1,2′[{f⊥},{f⊥*}]∣0〉1{f⊥}∣0〉2{f⊥*} (29)

Here, ${\hat{U}}_{1,2}^{\prime }[f,f^{*}]$ is a Gaussian operator only acting on mode *f* of channel 1 and mode f* of channel 2, and ${\hat{U}}_{1,2}^{\prime }[\{f_{⊥}\},\{f_{⊥}^{*}\}]$ acts on all other orthogonal modes. Then, the heralded state in mode *f* on channel 1 is given by

∣ψ〉1f=〈n∣2f*Uˆ1,2′[f,f*]∣0〉1f∣0〉2f* (30)

Thus, we can generate a non-Gaussian state ∣ϕ〉 in a complex mode *f*.

We derive Eq. 29 in the following. The operator ${\hat{Q}}^{†}[r]$ satisfies

$$\begin{array}{c} \left[{\hat{a}}_{1}[f],{\hat{Q}}^{†}[r]]=[\int \tilde{f}(-\omega ){\hat{\tilde{a}}}_{1}(\omega )\;d\omega ,\int \tilde{r}(\omega \prime ){\hat{\tilde{a}}}_{1}^{†}(\omega \prime ){\hat{\tilde{a}}}_{2}^{†}(-\omega \prime )\;d\omega \prime \right]\\ =\int \tilde{f}(\omega )\tilde{r}(\omega ){\hat{\tilde{a}}}_{2}^{†}(\omega )\;d\omega \\ =\langle \tilde{f}(\omega ),\tilde{f}(\omega )\tilde{r}(\omega )\rangle {\hat{a}}_{2}^{†}[f^{*}]\\ +\sqrt{{\left\|\tilde{f}(\omega )\tilde{r}(\omega )\right\|}^{2}-{|\langle \tilde{f}(\omega ),\tilde{f}(\omega )\tilde{r}(\omega )\rangle |}^{2}}\;{\hat{a}}_{2}^{†}[f_{⊥}^{*}]\\ =\|f^{*}r\|(\sqrt{M\prime [f,r]}\;{\hat{a}}_{2}^{†}[f^{*}]+\sqrt{1-M\prime [f,r]}\;{\hat{a}}_{2}^{†}[f_{⊥}^{*}]) \end{array}$$ (31)

where *M*′[*f*, *r*] = ∣〈*f*, *N*(f*r)〉∣^2^. Thus, the decomposition of ${\hat{Q}}^{†}[r]$ corresponding to Eq. 24 is given by

$$\begin{array}{c} {\hat{Q}}^{†}[r]=\|f^{*}r\|(\sqrt{M\prime [f,r]}\;{\hat{a}}_{1}^{†}[f]{\hat{a}}_{2}^{†}[f^{*}]+\sqrt{1-M\prime [f,r]}\;{\hat{a}}_{1}^{†}[f]{\hat{a}}_{2}^{†}[f_{⊥}^{*}])+\\ \left(\text{irrelevant terms}\right) \end{array}$$ (32)

The irrelevant terms always commute with ${\hat{a}}_{1}[f]$. We can show that the irrelevant terms also commute with ${\hat{a}}_{2}[f^{*}]$ when *M*′[*f*, *r*] = 1 considering decomposition of ${\hat{Q}}^{†}[r]$ based on a commutation relation $\left[{\hat{a}}_{2}[f^{*}],{\hat{Q}}^{†}[r]\right]$. When *M*′[*f*, *r*] = 1, ${\hat{T}}_{1,2}[r]$ is given by a direct product of an operator acting on mode *f* of channel 1 and mode f* of channel 2 as well as an operator acting on other modes. Because the decomposition of displacement operation in Eq. 23 is possible even for complex mode functions, we can decompose ${\hat{D}}_{1}(\alpha_{1},\omega_{d}){\hat{D}}_{2}(\alpha_{2},\omega_{d}){\hat{T}}_{1,2}[r]$ and get Eq. 23.

### Detail of the experiment

**Fig. 4.**
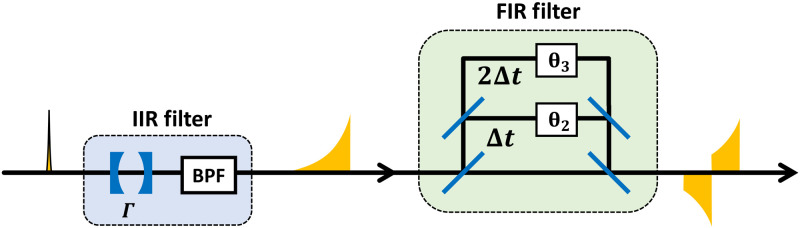
The diagram of the filter in photon measurement channel. IIR, infinite impulse response; FIR, finite impulse response; BPF, band-pass filter.

Figure 4 is the diagram of the filter we used in the experiment. This filter consists of an infinite impulse response (IIR) filter and a finite impulse response (FIR) filter. The IIR filter consists of an optical cavity resonant at ω = 0 and a band-pass filter to omit the resonant modes of the cavity at sidebands. Supposing the half width at half maximum (HWHM) of the cavity is Γ and the bandwidth of the band-pass filter is sufficiently broad, this step has a low pass–type impulse response given by

$$g_{\text{IIR}}(t)=\{\begin{array}{cc} 0 & \left(t<0\right)\\ \text{Γexp}\;[-\Gamma t] & \left(t\geq 0\right) \end{array}$$ (33)

The FIR filter is an interferometer with three arms. Each arm has a time delay 0, Δ*t*, and 2Δ*t*. The impulse response of this step is given by

$$g_{\text{FIR}}(t)=\kappa_{1}\delta (t)+\kappa_{2}e^{i\theta_{2}}\delta (t-\Delta t)+\kappa_{3}e^{i\theta_{3}}\delta (t-2\Delta t)$$ (34)

We can realize arbitrary *g*_FIR_(*t*) in the form of Eq. 34 up to a multiplication of a constant. Supposing κ_1_ : κ_2_ : κ_3_ = 1 : *e*^−ΓΔ*t*^ : 0 and θ_1_ = π, we get the desired impulse response given by

$$g_{\text{TB}}(t)=\{\begin{array}{cc} 0 & \left(t<0,\Delta t\leq t\right)\\ \kappa_{1}\text{Γexp}\;[-\Gamma t] & \left(0\leq t<\Delta t\right) \end{array}$$ (35)

We can also realize *g*_BTB_(*t*) given by

$$g_{\text{BTB}}(t)=({g_{\text{IIR}}}^{*}g_{\text{FIR}})(t)=\{\begin{array}{cc} 0 & \left(t<0,2\Delta t\leq t\right)\\ \kappa_{1}\Gamma \text{exp}\;[-\Gamma t] & \left(0\leq t<\Delta t\right)\\ -\kappa_{1}\Gamma \text{exp}\;[-\Gamma (t-\Delta t)] & \left(\Delta t\leq t<2\Delta t\right) \end{array}$$ (36)

where we put κ_1_ : κ_2_ : κ_3_ = 1 : 2*e*^−ΓΔ*t*^ : *e*^−2ΓΔ*t*^ and θ_2_ = π, θ_3_ = 0.

**Fig. 5.**
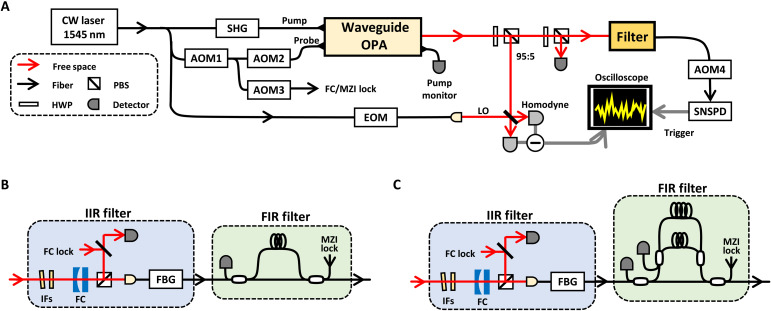
The detailed diagram of experimental setup. (**A**) Whole setup. AOM, acousto-optic modulator; SHG, second harmonic generation; EOM, electro-optic modulator; PBS, polarization beam splitter; HWP, half wave plate. (**B**) The filter for state generation in a TB mode. IF, interference filter; FC, filter cavity; MZI, Mach-Zehnder interferometer. (**C**) The filter for state generation in a BTB mode.

Figure 5 shows the whole setup of the experiment. The main light source is a CW laser at 1545 nm. The CW pump beam is generated at a second-harmonic generation module. The IIR part of the filter consists of a Fabry-Pérot cavity (HWHM = 8.2 MHz, FSR = 8.5 GHz), an interferometric filter (HWHM = 130 GHz), and a fiber Bragg grating (HWHM = 3.6 GHz). The FIR part is a fiber-based interferometer. From Δ*t* = 20 ns and Γ = 2π × 8.2 MHz, we calculate the ratio of κ*_i_* (*i* = 1,2,3). We equally distribute the input light to the short, medium, and long arms in the case of the BTB mode and to the short and medium arms in the case of the TB mode. Then, we adjust κ*_i_* to the desired ratio by the variable directional coupler at the output side. The SNSPD is installed into an adiabatic demagnetization refrigerator with an operational temperature around 500 mK. A detection efficiency and a dark count are 63% and 100 counts per second (cps), respectively. We use probe light and lock light as the phase reference of the heralded state and the filter elements, respectively. The phases are locked via piezo elements attached to mirrors and fibers. Probe and lock lights are turned off during the measurement of heralded states because they easily saturate the SNSPD. We use acousto-optic modulators (AOMs) to switch between the control phase and the measurement phase with a period of 1.6 kHz. In the control phase, AOMs 1 to 3 in Fig. 5 are open, but AOM4 is closed. The opposite is true in the measurement phase.

In the TB mode experiment, the pump light power is 15 mW, the LO light power is 5 mW, and the SNSPD count is 21.0 kilo cps (kcps), of which the fake count is 400 cps. In the BTB mode experiment, the pump light power is 12 mW, the LO light power is 10 mW, and the SNSPD count is 4.2 kcps, of which the fake count is 200 cps. Because the transmittance of the filters is not optimized in this experiment, the state generation rate is not very high especially in the case of the BTB mode. In principle, it is possible to make a filter with 100% transmittance for any impulse response (*48*), so this point can be much improved in the future. The large fake count in the TB mode is due to the stray light caused by the LO light. It does not matter in the case of the BTB mode because the interferometer removes the stray light. The uncertainties of the analysis results are calculated by using bootstrapping method (*59*).

## References

[1] S. T. Cundiff, A. M. Weiner, Optical arbitrary waveform generation. Nat. Photonics 4, 760–766 (2010).

[2] A. M. Weiner, Ultrafast optical pulse shaping: A tutorial review. Opt. Commun. 284, 3669–3692 (2011).

[3] K.-K. Ni, S. Ospelkaus, M. H. G. de Miranda, A. Pe’er, B. Neyenhuis, J. J. Zirbel, S. Kotochigova, P. S. Julienne, D. S. Jin, J. Ye, A high phase-space-density gas of polar molecules. Science 322, 231–235 (2008). 18801969 10.1126/science.1163861

[4] M. C. Stowe, F. C. Cruz, A. Marian, J. Ye, High resolution atomic coherent control via spectral phase manipulation of an optical frequency comb. Phys. Rev. Lett. 96, 153001 (2006). 16712153 10.1103/PhysRevLett.96.153001

[5] I. Morichika, K. Murata, A. Sakurai, K. Ishii, S. Ashihara, Molecular ground-state dissociation in the condensed phase employing plasmonic field enhancement of chirped mid-infrared pulses. Nat. Commun. 10, 3893 (2019). 31467268 10.1038/s41467-019-11902-6 PMC6715752

[6] D. J. Geisler, N. K. Fontaine, T. He, R. P. Scott, L. Paraschis, J. P. Heritage, S. J. B. Yoo, Modulation-format agile, reconfigurable Tb/s transmitter based on optical arbitrary waveform generation. Opt. Express 17, 15911–15925 (2009). 19724590 10.1364/OE.17.015911

[7] D. J. Geisler, N. K. Fontaine, R. P. Scott, T. He, L. Paraschis, O. Gerstel, J. P. Heritage, S. J. B. Yoo, Bandwidth scalable, coherent transmitter based on the parallel synthesis of multiple spectral slices using optical arbitrary waveform generation. Opt. Express 19, 8242–8253 (2011). 21643074 10.1364/OE.19.008242

[8] S. Cundiff, S. Mukamel, Optical multidimensional coherent SPECTROSCOPY. Phys. Today 66, 44–49 (2013).

[9] F. D. Fuller, D. E. Wilcox, J. P. Ogilvie, Pulse shaping based two-dimensional electronic spectroscopy in a background free geometry. Opt. Express 22, 1018–1027 (2014). 24515061 10.1364/OE.22.001018

[10] J. Nokkala, F. Arzani, F. Galve, R. Zambrini, S. Maniscalco, J. Piilo, N. Treps, V. Parigi, Reconfigurable optical implementation of quantum complex networks. New J. Phys. 20, 053024 (2018).

[11] J. L. O’Brien, Optical quantum computing. Science 318, 1567–1570 (2007). 18063781 10.1126/science.1142892

[12] S. Takeda, A. Furusawa, Toward large-scale fault-tolerant universal photonic quantum computing. APL Photonics 4, 060902 (2019).

[13] N. Gisin, G. Ribordy, W. Tittel, H. Zbinden, Quantum cryptography. Rev. Mod. Phys. 74, 145–195 (2002).

[14] N. Gisin, R. Thew, Quantum communication. Nat. Photonics 1, 165–171 (2007).

[15] H. J. Kimble, The quantum internet. Nature 453, 1023–1030 (2008). 18563153 10.1038/nature07127

[16] K. Fukui, R. N. Alexander, P. van Loock, All-optical long-distance quantum communication with Gottesman-Kitaev-Preskill qubits. Phys. Rev. Res. 3, 033118 (2021).

[17] J. Abadie, B. P. Abbott, R. Abbott, T. D. Abbott, M. Abernathy, C. Adams, R. Adhikari, C. Affeldt, P. Ajith, B. Allen, G. S. Allen, E. A. Ceron, D. Amariutei, R. S. Amin, S. B. Anderson, W. G. Anderson, K. Arai, M. A. Arain, M. C. Araya, S. M. Aston, D. Atkinson, P. Aufmuth, C. Aulbert, B. E. Aylott, S. Babak, P. Baker, S. Ballmer, D. Barker, B. Barr, P. Barriga, S. Ghosh, A gravitational wave observatory operating beyond the quantum shot-noise limit. Nat. Phys. 7, 962–965 (2011).

[18] J. Yoshikawa, S. Yokoyama, T. Kaji, C. Sornphiphatphong, Y. Shiozawa, K. Makino, A. Furusawa, Invited Article: Generation of one-million-mode continuous-variable cluster state by unlimited time-domain multiplexing. APL Photonics 1, 060801 (2016).

[19] M. V. Larsen, X. Guo, C. R. Breum, J. S. Neergaard-Nielsen, U. L. Andersen, Deterministic generation of a two-dimensional cluster state. Science 366, 369–372 (2019). 31624213 10.1126/science.aay4354

[20] W. Asavanant, Y. Shiozawa, S. Yokoyama, B. Charoensombutamon, H. Emura, R. N. Alexander, S. Takeda, J. Yoshikawa, N. C. Menicucci, H. Yonezawa, A. Furusawa, Generation of time-domain-multiplexed two-dimensional cluster state. Science 366, 373–376 (2019). 31624214 10.1126/science.aay2645

[21] M. V. Larsen, X. Guo, C. R. Breum, J. S. Neergaard-Nielsen, U. L. Andersen, Deterministic multi-mode gates on a scalable photonic quantum computing platform. Nat. Phys. 17, 1018–1023 (2021).

[22] Y. Enomoto, K. Yonezu, Y. Mitsuhashi, K. Takase, S. Takeda, Programmable and sequential Gaussian gates in a loop-based single-mode photonic quantum processor. Sci. Adv. 7, eabj6624 (2021). 34767450 10.1126/sciadv.abj6624 PMC8589304

[23] J. Yoshikawa, M. Bergmann, P. van Loock, M. Fuwa, M. Okada, K. Takase, T. Toyama, K. Makino, S. Takeda, A. Furusawa, Heralded creation of photonic qudits from parametric down-conversion using linear optics. Phys. Rev. A 97, 053814 (2018).

[24] H. Ogawa, H. Ohdan, K. Miyata, M. Taguchi, K. Makino, H. Yonezawa, J. Yoshikawa, A. Furusawa, Real-time quadrature measurement of a single-photon wave packet with continuous temporal-mode matching. Phys. Rev. Lett. 116, 233602 (2016). 27341231 10.1103/PhysRevLett.116.233602

[25] J. T. Barreiro, N. K. Langford, N. A. Peters, P. G. Kwiat, Generation of hyperentangled photon pairs. Phys. Rev. Lett. 95, 260501 (2005). 16486324 10.1103/PhysRevLett.95.260501

[26] D. E. Bruschi, S. Chatzinotas, F. K. Wilhelm, A. W. Schell, Spacetime effects on wavepackets of coherent light. Phys. Rev. D 104, 085015 (2021).

[27] M. Stobińska, G. Alber, G. Leuchs, Perfect excitation of a matter qubit by a single photon in free space. Europhys. Lett. 86, 14007 (2009).

[28] F. Khalili, S. Danilishin, H. Miao, H. Müller-Ebhardt, H. Yang, Y. Chen, Preparing a mechanical oscillator in non-gaussian quantum states. Phys. Rev. Lett. 105, 070403 (2010). 20868023 10.1103/PhysRevLett.105.070403

[29] J. Nunn, I. A. Walmsley, M. G. Raymer, K. Surmacz, F. C. Waldermann, Z. Wang, D. Jaksch, Mapping broadband single-photon wave packets into an atomic memory. Phys. Rev. A 75, 011401 (2007).

[30] J. Yoshikawa, K. Makino, S. Kurata, P. van Loock, A. Furusawa, Creation, storage, and on-demand release of optical quantum states with a negative wigner function. Phys. Rev. X 3, 041028 (2013).

[31] G. Vittorio, L. Seth, M. Lorenzo, Quantum-enhanced measurements: Beating the standard quantum limit. Science 306, 1330–1336 (2004). 15550661 10.1126/science.1104149

[32] M. Ohliger, K. Kieling, J. Eisert, Limitations of quantum computing with Gaussian cluster states. Phys. Rev. A 82, 042336 (2010).

[33] A. Mari, J. Eisert, Positive wigner functions render classical simulation of quantum computation efficient. Phys. Rev. Lett. 109, 230503 (2012). 23368175 10.1103/PhysRevLett.109.230503

[34] M. Yukawa, K. Miyata, T. Mizuta, H. Yonezawa, P. Marek, R. Filip, A. Furusawa, Generating superposition of up-to three photons for continuous variable quantum information processing. Opt. Express 21, 5529–5535 (2013). 23482124 10.1364/OE.21.005529

[35] W. Asavanant, K. Nakashima, Y. Shiozawa, J. Yoshikawa, A. Furusawa, Generation of highly pure Schrödinger’s cat states and real-time quadrature measurements via optical filtering. Opt. Express 25, 32227–32242 (2017).

[36] S. Takeda, H. Benichi, T. Mizuta, N. Lee, J. Yoshikawa, A. Furusawa, Quantum mode filtering of non-Gaussian states for teleportation-based quantum information processing. Phys. Rev. A 85, 053824 (2012).

[37] K. Takase, M. Okada, T. Serikawa, S. Takeda, J. Yoshikawa, A. Furusawa, Complete temporal mode characterization of non-Gaussian states by a dual homodyne measurement. Phys. Rev. A 99, 033832 (2019).

[38] L. S. Costanzo, A. S. Coelho, D. Pellegrino, M. S. Mendes, L. Acioli, K. N. Cassemiro, D. Felinto, A. Zavatta, M. Bellini, Zero-area single-photon pulses. Phys. Rev. Lett. 116, 023602 (2016). 26824539 10.1103/PhysRevLett.116.023602

[39] Y.-S. Ra, A. Dufour, M. Walschaers, C. Jacquard, T. Michel, C. Fabre, N. Treps, Non-Gaussian quantum states of a multimode light field. Nat. Phys. 16, 144–147 (2020).

[40] X. Wang, L. Zhou, R. Li, J. Xie, L. Lu, K. Wu, J. Chen, Continuously tunable ultra-thin silicon waveguide optical delay line. Optica 4, 507–515 (2017).

[41] T. B. Pittman, B. C. Jacobs, J. D. Franson, Single photons on pseudodemand from stored parametric down-conversion. Phys. Rev. A 66, 042303 (2002).

[42] F. Kaneda, B. G. Christensen, J. J. Wong, H. S. Park, K. T. McCusker, P. G. Kwiat, Time-multiplexed heralded single-photon source. Optica 2, 1010–1013 (2015).

[43] I. Tzitrin, J. E. Bourassa, N. C. Menicucci, K. K. Sabapathy, Progress towards practical qubit computation using approximate Gottesman-Kitaev-Preskill codes. Phys. Rev. A 101, 032315 (2020).

[44] K. Takase, J. Yoshikawa, W. Asavanant, M. Endo, A. Furusawa, Generation of optical Schrödinger cat states by generalized photon subtraction. Phys. Rev. A 103, 013710 (2021).

[45] T. Kashiwazaki, T. Yamashima, N. Takanashi, A. Inoue, T. Umeki, A. Furusawa, Fabrication of low-loss quasi-single-mode PPLN waveguide and its application to a modularized broadband high-level squeezer. Appl. Phys. Lett. 119, 251104 (2021).

[46] K. Takase, A. Kawasaki, B. K. Jeong, M. Endo, T. Kashiwazaki, T. Kazama, K. Enbutsu, K. Watanabe, T. Umeki, S. Miki, H. Terai, M. Yabuno, F. China, W. Asavanant, J. Yoshikawa, A. Furusawa, Generation of Schrödinger cat states with Wigner negativity using a continuous-wave low-loss waveguide optical parametric amplifier. Opt. Express 30, 14161–14171 (2022). 35473166 10.1364/OE.454123

[47] R. Shimazu, N. Yamamoto, Quantum functionalities via feedback amplification. Phys. Rev. Applied 15, 044006 (2021).

[48] K. Jinguji, Synthesis of coherent two-port optical delay-line circuit with ring waveguides. J. Light. Technol. 14, 1882–1898 (1996).

[49] M. Dakna, T. Anhut, T. Opatrný, L. Knöll, D.-G. Welsch, Generating Schrödinger-cat-like states by means of conditional measurements on a beam splitter. Phys. Rev. A 55, 3184–3194 (1997).

[50] S. Miki, M. Yabuno, T. Yamashita, H. Terai, Stable, high-performance operation of a fiber-coupled superconducting nanowire avalanche photon detector. Opt. Express 25, 6796–6804 (2017). 28381022 10.1364/OE.25.006796

[51] O. Morin, C. Fabre, J. Laurat, Experimentally accessing the optimal temporal mode of traveling quantum light states. Phys. Rev. Lett. 111, 213602 (2013). 24313487 10.1103/PhysRevLett.111.213602

[52] A. I. Lvovsky, M. G. Raymer, Continuous-variable optical quantum-state tomography. Rev. Mod. Phys. 81, 299–332 (2009).

[53] D. Pérez, I. Gasulla, L. Crudgington, D. J. Thomson, A. Z. Khokhar, K. Li, W. Cao, G. Z. Mashanovich, J. Capmany, Multipurpose silicon photonics signal processor core. Nat. Commun. 8, 636 (2017). 28935924 10.1038/s41467-017-00714-1 PMC5608755

[54] N. Takanashi, A. Inoue, T. Kashiwazaki, T. Kazama, K. Enbutsu, R. Kasahara, T. Umeki, A. Furusawa, All-optical phase-sensitive detection for ultra-fast quantum computation. Opt. Express 28, 34916–34926 (2020). 33182949 10.1364/OE.405832

[55] B. Korzh, Q. Y. Zhao, J. P. Allmaras, S. Frasca, T. M. Autry, E. A. Bersin, A. D. Beyer, R. M. Briggs, B. Bumble, M. Colangelo, G. M. Crouch, A. E. Dane, T. Gerrits, A. E. Lita, F. Marsili, G. Moody, C. Peña, E. Ramirez, J. D. Rezac, N. Sinclair, M. J. Stevens, A. E. Velasco, V. B. Verma, E. E. Wollman, S. Xie, D. Zhu, P. D. Hale, M. Spiropulu, K. L. Silverman, R. P. Mirin, S. W. Nam, A. G. Kozorezov, M. D. Shaw, K. K. Berggren, Demonstration of sub-3 ps temporal resolution with a superconducting nanowire single-photon detector. Nat. Photonics 14, 250–255 (2020).

[56] J. Roslund, R. M. de Araújo, S. Jiang, C. Fabre, N. Treps, Wavelength-multiplexed quantum networks with ultrafast frequency combs. Nat. Photonics 8, 109–112 (2014).

[57] P. P. Rohde, W. Mauerer, C. Silberhorn, Spectral structure and decompositions of optical states, and their applications. New J. Phys. 9, 91 (2007).

[58] J. Yoshikawa, W. Asavanant, A. Furusawa, Purification of photon subtraction from continuous squeezed light by filtering. Phys. Rev. A 96, 052304 (2017).

[59] B. Efron, R. J. Tibshirani, *An introduction to the bootstrap* (CRC press, 1994).

